# Assessment during clinical education among nursing students using two different assessment instruments

**DOI:** 10.1186/s12909-024-05771-x

**Published:** 2024-08-07

**Authors:** Nilsson Tomas, Masiello Italo, Broberger Eva, Lindström Veronica

**Affiliations:** 1 Department of Clinical Science and Education, Södersjukhuset, Karolinska Institutet, 11883 Stockholm, Sweden; 2 Department of Pedagogy, Linnaeus University, Växjö, Sweden; 3 Department for Neurobiology, Care Sciences, and Society, Division of Nursing, Karolinska Institutet, Huddinge, Sweden; 4 Department of Computer Science and Media Technology, Linnaeus University, Växjö, Sweden; 5 Department of Nursing, Division of Ambulance Service, Region Västerbotten, Umeå University, Umeå, Sweden; 6 Department of Health Promotion Science, Sophiahemmet University, Stockholm, Sweden

**Keywords:** Assessment, Clinical education, Feedback, Learning objectives

## Abstract

**Background:**

Assessment of undergraduate students using assessment instruments in the clinical setting is known to be complex. The aim of this study was therefore to examine whether two different assessment instruments, containing learning objectives (LO`s) with similar content, results in similar assessments by the clinical supervisors and to explore clinical supervisors’ experiences of assessment regarding the two different assessment instruments.

**Method:**

A mixed-methods approach was used. Four simulated care encounter scenarios were evaluated by 50 supervisors using two different assessment instruments. 28 follow-up interviews were conducted. Descriptive statistics and logistic binary regression were used for quantitative data analysis, along with qualitative thematic analysis of interview data.

**Result:**

While significant differences were observed within the assessment instruments, the differences were consistent between the two instruments, indicating that the quality of the assessment instruments were considered equivalent. Supervisors noted that the relationship between the students and supervisors could introduce subjectivity in the assessments and that working in groups of supervisors could be advantageous. In terms of formative assessments, the Likert scale was considered a useful tool for evaluating learning objectives. However, supervisors had different views on grading scales and the need for clear definitions. The supervisors concluded that a complicated assessment instrument led to limited very-day usage and did not facilitate formative feedback. Furthermore, supervisors discussed how their experiences influenced the use of the assessment instruments, which resulted in different descriptions of the experience. These differences led to a discussion of the need of supervisor teams to enhance the validity of assessments.

**Conclusion:**

The findings showed that there were no significant differences in pass/fail gradings using the two different assessment instruments. The quantitative data suggests that supervisors struggled with subjectivity, phrasing, and definitions of the LO´s and the scales used in both instruments. This resulted in arbitrary assessments that were time-consuming and resulted in limited usage in the day-to-day assessment. To mitigate the subjectivity, supervisors suggested working in teams and conducting multiple assessments over time to increase assessment validity.

**Supplementary Information:**

The online version contains supplementary material available at 10.1186/s12909-024-05771-x.

## Introduction

During undergraduate studies to become a registered nurse (RN), the assessment of clinical competence includes an assessment of both theoretical knowledge and practical skills [28]. The importance of high-quality clinical education for students that provides constructive and adapted feedback is a key factor affecting the student’s learning process and should not be understated [28, 30]. Historically, different assessment instruments have been designed aiming both to support the clinical supervisors and to provide standardised, fair assessments of students’ achievement of the learning objectives (LOs) [10]. However, the assessment of nursing students’ skills and competence using assessment instruments is known to be complex and affected by several varied factors [29]. The assessment instrument, Assessment of Clinical Education (ACIEd), that is used in this study context, is used in a summative fashion to verify that the students earn a passing grade during clinical education. Formative assessment aims to support the students through continuous feedback. To support the student’s development towards becoming a RN, an assessment instrument that facilitates both summative and formative assessment is needed [1].

## Background

A well-educated RN is essential to achieve good health care. To realise this, supervisors, as well as students, need support during clinical education in facilitating student learning [12]. Nevertheless, supporting, and supervising students is known to be complex since learning in the clinical setting is affected by several factors, such as the clinical context, the student’s own strengths, workplace challenges, expectations, and the student’s social network and prior knowledge and skills [3, 4]. For the students to succeed in their learning, an un-bias assessment with LO`s that are relevant for the student’s progression is necessary.

### Assessment in clinical education

Assessment is a vital part of clinical education and should include both skills and theoretical knowledge reflecting the requirements of the RN everyday work and having a close connection to the university curriculum [17]. The assessment of nursing students can be accomplished in a formative or summative way. The formative assessment is meant to guide the nursing students in their learning progress and is a joint discussion between the student and the supervisor where strengths and areas of improvement are identified and addressed [6]. The summative assessment’s prime objective is to ensure that LOs are achieved, and it commonly occurs on one or two occasions during a period/session of clinical education with a university teacher present [15]. However, clinical assessment is sometimes hampered by the clinical supervisors’ lack of training in assessing students’ nursing skills and competence and/or the supervisors’ lack of competence in using a standard assessment instrument [17]. In addition, a heavy workload and staff shortages in the clinical setting make it difficult for the clinical supervisors to find time to assess students properly. It is also known that the relationship between supervisor and student can affect the assessment [11]. To aid the assessment of students’ nursing skills and competence, assessment instruments need to be reliable, valid, easy to use and adapted to the clinical setting [7]. Today, several different assessment instruments exist which all have strengths and weaknesses and focus on various aspects of the student’s learning. These instruments utilize different approaches to assessment where for example Observed Structural Clinical Examination (OSCE) uses checklists and Assessment of Clinical Education (ACIEd) uses more complex LO`s that require subjective assessments [22, 26, 27]. ACIEd, is used by several universities in Sweden for assessment of LO`s during clinical education. The ACIEd was designed for a mid and final assessment, where the mid assessment results in: “In line with expected achievement” or “Plan of action is needed”. The final assessment results in a “Pass” or “Fail” grade on each LO. In clinical education, every LO needs to be passed to get a final pass grade [26, 27]. Criticism has been raised in the ambulance services in Stockholm, Sweden, towards ACIEd, claiming that it is ill adapted for daily use and has a summative character where progression is hard to visualize. Therefore, a new assessment instrument was developed from the ACIEd and named the Ambulance Assessment Instrument (AAI). The intent of the construction of AAI was to provide alternatives to the existing assessment instrument that was designed for digital use in the clinical setting.

The AAI was constructed with more distinct and phrased LOs listed one by one, instead of using complex LOs with several sub-LOs imbedded in one. A seven graded Likert scale facilitated formative assessment. The rationale for clarifying LOs was that earlier research has shown that complex LOs lead to interpretations and inconsistencies in assessment [17]. Therefore, this was considered when the AAI was developed, resulting in one-sentence LOs without sub-criteria. The assessment instruments can be found in appendix 1. However, to incorporate a new assessment instrument in clinical education, it needs to be validated. In this case the research group choose to validate the developed AAI instrument against the existing instrument ACIEd. The aim of this study was therefore to examine whether two different assessment instruments, containing LO´s with similar content, results in similar assessments by the clinical supervisors and to explore clinical supervisors’ experiences of assessment regarding the two different assessment instruments.

### Clinical education in the ambulance service

The clinical setting in this study was the ambulance services in Stockholm, Sweden. Care in an ambulance is characterised by short patient encounters, ever-changing environments, and patients seeking care for all varieties of complaints [19, 25, 31]. Typical is also a lack of preparation time and little or no chance for the students to pause and step out of the care encounter to discuss strategies or reflect on care situations that have occurred. The ambulance service has similar challenges to those faced in ambulatory care [9]. Historically, the ambulance service is an uncommon placement for clinical education in undergraduate nursing education due to the environment and lack of RNs working in the service. At present, every ambulance in Sweden is staffed by at least one RN [16], and it is stated that nursing students have the possibility to learn nursing skills in the ambulance services [17, 18, 20, 21]. In the region of Stockholm, the nurses have at least one year of additional training which, for example, could be prehospital care, anesthesiology, emergency care, psychiatric care, or midwifery. The amount of work experience can vary greatly. Among the employed nurses its almost an even split between males and females.

## Material and method

A mixed-method study design was used [23]. Quantitative data was generated from supervisors when assessing pre-recorded, simulated patient encounters performed by students. Individual interviews, conducted directly after the assessments, were completed in accordance with a mixed-method design.

### Assessment instruments

The ACIEd used in this study consists of five LOs with sub-criteria concerning what the student needs to achieve to receive a Pass grade during a course in emergency care. The LOs are designed to cover all aspects of the six-week clinical education in relation to the course objectives resulting in LOs with several goals embedded in one, for example, LO 1, which translated reads:

1. Approach and support patients and their relatives in respectful consultation and perform nursing care based on the patient's experience of the situation.

To clarify this LO and reduce the risk of inconsistencies in assessment one-sentence LOs without sub-criteria was reconstructed in the AAI, and one example of this reads as follows:

- 1.1. To what extent was the patient treated with respect?
- 1.2. To what extent was the patient allowed to describe his or her situation?
- 1.3. To what extent did the student create a safe care situation?

The complete list of LO´s used can be found in appendix one. To enable formative assessment by displaying progress using the assumption that students’ performances will generate higher grades as their clinical education progresses, the AAI has a seven-point Likert scale with a pass grade marked as 5, meaning that scores 1 to 4 results in a fail grade. The Likert scale was given descriptions from 1 = “*Not at all”* to 7 = “*To a great extent”.* Furthermore, the ACIEd separates midterm assessment from final assessment, where grades in midterm are referred to as “In line with objective” or “Plan of action is required”. The final assessment using ACIEd results in a pass or fail grade. The AAI provides a formative assessment, which is meant to be repeated frequently but can also act as a basis for grading in an equivalent way as the ACIEd.

### Simulated patient encounters

Four different recorded simulated scenarios were used in the study (recorded time: 6min 58 s, 3min 14s, 5min 58s, 2min 32 s). The scenarios had variations of student performances with the intention of generating variations in supervisor assessments. Differences were seen in both students’ assessments of the patient and in their treatment strategies. Two scenarios included a student interacting with a standardised patient, while two scenarios had a student interacting with a patient simulator manikin. The standardised patient was a middle-aged woman with fatigue and dizziness. The patient simulator manikin was a young man with abdominal pain. The nursing students participating in the simulation scenarios were recruited from the fifth semester of the nursing study programme at one university in Stockholm, Sweden, after they had finished their six-week clinical education in the ambulance services and all grades had been made official. Both male and female students participated in the simulations.

### Participants

A convenience sample of 50 clinical supervisors, all RN in the ambulance service, participated in the study, having varying experience of supervisorship. The participants were recruited at three different emergency departments by the main author. By selecting different emergency departments, it was possible to include participants with different ambulance service employers (private and public). The clinical supervisors from the ambulance service were asked to participate in the study after they arrived at the emergency department and the hand-over of the patient was completed. The convenience sampling of clinical supervisors was used due to the difficulty in recruiting participants at the ambulance station, as they were constantly mobile during their shifts. The supervisors were provided with both written and oral information about the study as well as a letter of consent for participation in the study. No participants were excluded due to experience, gender, educational level, or other factors.

### Data collection

In total, 50 clinical supervisors from the ambulance service assessed and graded four recorded simulations in accordance with the LO described in the ACIEd and the AAI instrument. No supervisor declined the offer to participate in this part of the study. Before the assessment started, a randomisation process was conducted. Firstly, the order of the simulations was randomised using a lottery (standardised patient vs. patient simulator manikin.). Secondly, the two scenarios were randomised using lottery (scenarios 1 and 2). No power calculations were performed. The recorded simulations were watched in one sequence without time to reflect or discuss the assessment and grading with others. All supervisors had prior knowledge of the assessment instrument ACIEd but had never used AAI.

The qualitative data consisted of interviews, conducted after the participants had assessed the simulated patient encounters. The interviews started with the open question “What are your thoughts about the assessment instruments?” Probing questions were then used to explore the participants’ experience of using the assessment instruments when grading the student's performance in the recorded simulations. The interviews varied in length from a few minutes up to 30 min. In total, 28 interviews were conducted (Female: 11 Male: 17). Fieldnotes were used during the interviews and after every finished interview the fieldnotes were summarized and reviewed. Theses reviews were used to make changes to the probing questions in relations to the aim of the study. In 22 cases there were no interviews conducted due to participants’ shortage of time. The supervisors were not informed about the questions prior to the interview.

### Data analysis

The data was analysed in two parts, a quantitative and qualitative. The quantitative data was analysed by compiling the assessments generated by the two assessment instruments and compared in a simple figure where the difference was described from the perspective of how many pass grades the assessment instrument generated. Secondly, a logistic binary regression analysis was used to examine whether the grades generated by two different assessment instruments was affected by the supervisor’s gender and/or experience. To explore whether work experience as an RN affected the grading of student’s performance, a dichotomisation of the work experience variable was carried out. The variables were dichotomised into $\le$ 6 and 6 > years of working experience as RNs (Dichotomised 1 for $\le$ 6 and 2 for 6 >). This dichotomisation was based on the theory by Benner, that experienced nurses can use their experience, knowledge, and additional perspectives instead of relying on standardised guidelines, tests, and regulations [2] to assess the students according to the LO`s. A gender dichotomisation was also performed to investigate if there was any difference in grading related to gender which was defined as male or female with no consideration to other gender definitions (Gender was coded 1 for males and 2 for females). This dichotomisation was based on the diversity of the staff in the ambulance service. The independent variables for both instruments were coded as 1 for a pass grade and 2 for fail. After considering the number of included supervisors in the study the p value was set to 0.05. The data was analysed using Statistical Package for the Social Sciences, version 24, Chicago, IL, USA in combination with Microsoft Excel 2010 (Microsoft Corp, Richmond, WA, USA).

The interviews were analysed using a thematic analysis approach [5]. The method was chosen due to its flexible nature. The themes constructed was done in a “theory driven way” meaning that the research question was clearly present in the coding in contrast to an inductive analysis approach. First, in the analysis the field notes were read several times to gain familiarity with the content. Secondly, codes were identified that described clinical supervisors’ experience of using the two different assessment instruments were identified. Thirdly, the codes were examined, and by identifying broader patterns of meaning, potential themes were constructed. Fourthly, a thematic map was constructed, and the themes were checked on two levels. First the codes were checked against the theme making sure that the codes formed a coherent pattern. In the second level the themes were checked against the entire dataset and in relations to the other themes to ensure that the themes did not intertwine with each other and finally, the themes were named.

In the fifth step the themes and sub-themes were related back to the narrative making sure that the themes captured the full story and that each theme was unique and contained valid information. The “story” that the themes and sub-themes were checked against, was the researcher’s contextual knowledge and experience. Lastly, the report was written up using the themes and the sub- themes. The first author initiated the analysis and the corresponding author participated in the analysis process.

## Quantitative results

In total, 34 (68%) male and 16 (32%) female clinical supervisors participated in the study. The work experience as an RN ranged from four months to 19 years, with a mean of 7.95 years. The work experience from the ambulance services ranged from 2.5 months to 35 years with a mean of 6.63 years (missing data n = 5). All participants had experience of clinical supervision and assessing nursing students during clinical education. The logistic binary regression analysis showed that there was no significant difference in 23 out of 24 LOs as displayed in Tables 1, 2, 3 and 4. One significant difference was found in the ACIEd LO 2 (*p* = 0.021) when the length of experience among the clinical supervisors was used as a dependent variable (Table 4). LO 2 assessed the students’ knowledge of the technical equipment and how they interacted with the patient while using the equipment.

**Table 1:** Effect of the dependent variable ‘Genders’ on grading related to learning objectives using a manikin for both the AAI and ACIEd with 1 degree of freedom

Independent variable LOs	Beta coefficient	Standard error	Wald chi-square test	*P*-Value	Exponentiation of the B coefficient
AAI^a^ LO1	0.451	0.866	0.271	0.602	1.571
AAI LO 2	-0.932	0.813	1.314	0.252	0.394
AAI LO 3	0.277	1.024	0.073	0.787	1.319
ACIEd^b^ LO 1	-0.478	0.960	0.247	0.619	0.620
ACIEd LO 2	0.969	0.965	1.009	0.315	2.636
ACIEd LO 3	-0.003	0.965	0.000	0.998	0.997

^a^*AAI* Ambulance Assessment Instrument

^b^*ACIEd* Assessment of Clinical Education

**Table 2:** Effect of the dependent variable ‘Experience’ on grading related to learning objectives using a manikin for both the AAI and ACIEd with 1 degree of freedom

Independent variable LOs	Beta coefficient	Standard error	Wald chi- square test	*P*-Value	Exponentiation of the B coefficient
AAI^a^ LO 1	0.600	1.117	0.288	0.591	1.822
AAI LO 2	2.418	1.279	3.574	0.059	11.219
AAI LO 3	0.167	1.306	0.016	0.898	1.182
ACIEd^b^ LO 1	1.346	1.186	1.288	0.256	3.841
ACIEd LO 2	-2.193	1.546	2.012	0.156	0.112
ACIEd LO 3	-1.955	1.220	2.568	0.109	0.142

^a^*AAI* Ambulance Assessment Instrument

^b^*ACIEd* Assessment of Clinical Education

**Table 3:** Effect of the dependent variable ‘Gender’ on grading related to learning objectives using the Standardized patient for both the AAI and ACIEd with 1 degree of freedom

Independent variable LOs	Beta coefficient	Standard error	Wald chi-square test	*P*-Value	Exponentiation of the B coefficient
AAI^a^ LO 1	0.499	1.021	0.239	0.625	1.647
AAI LO 2	0.561	0.597	0.883	0.347	1.752
AAI LO 3	0.540	0.825	0.428	0.513	1.716
ACIEd^b^ LO 1	-0.992	0.987	1.010	0.315	0.371
ACIEd LO 2	0.328	0.725	0.205	0.651	1.388
ACIEd LO 3	-0.329	1.092	0.091	0.763	0.720

^a^*AAI* Ambulance Assessment Instrument

^b^*ACIEd* Assessment of Clinical Education

**Table 4:** Effect of the dependent variable ‘Experience’ on grading related to Learning objectives using the Standardized patient for both the AAI and ACIEd with 1 degree of freedom

Independent variable LOs	Beta coefficient	Standard error	Wald chi-square test	*P*-Value	Exponentiation of the B coefficient
AAI* LO 1	-0.405	0.970	0.174	0.676	0.667
AAI LO 2	0.662	0.562	1.388	0.239	1.939
AAI LO 3	-0.527	0.788	0.447	0.504	0.590
ACIEd^a^ LO 1	-0.204	0.972	0.044	0.834	0.815
ACIEd LO 2	1.918	0.834	5.289	0.021*	6.805
ACIEd LO 3	-1.260	1.160	1.181	0.277	0.284

*AAI Ambulance Assessment Instrument

^a^*ACIEd* Assessment of Clinical Education

### Qualitative results

The thematic analysis used to explore clinical supervisors’ experience of assessment in relation to the two different assessment instruments resulted in three themes: *Learning objectives*, *Assessment* and *Supervisorship.*

#### Learning objectives

Supervisors described a variety of experiences relating to the LO`s in the two sub-themes: Phrasing of the LOs, subjectivity in the LOs, Complexity of the LO`s. The subthemes describe the supervisors’ view of the learning objectives in relation to how the LO`s are constructed and how they are interpreted as well as how they are used in the daily activities.

##### Phrasing of the learning objectives

The supervisors discussed the language used in the ACIEd to describe the LO`s and that the academic writing created confusion and was complex and hard to understand. They also discussed the lack of clear definitions, and difficulties using the ACIEd with several sub-criteria embedded in one LO, which resulted in individual interpretation of the LO and a risk of subjective assessment. According to the supervisors’ reasoning, several sub-criteria in the same LO complicated the assessment when students performed well according to some of the sub-criteria and poorly according to others. The supervisors expressed that the LOs used in AAI had a clearer phrasing making it easier to use. LO`s related to communication and patient relations were considered complicated to assess using the ACIEd in contrast to LO`s regarding medical procedures where right and wrong was clearly defined. Medical procedures were considered easier to assess in both instruments.

##### Complexity of the learning objective

When assessing the students’ nursing and care skills in the simulations, the supervisors claimed that the complexity of ACIEd made it challenging to explaining why they assessed as they did. In the clinic setting the supervisors said that the complexity of the LO`s made the usage of ACIEd time-consuming and poorly adapted to everyday work, which resulted in limited use, and only used in a summative way right before mid and end assessments. Altogether, the ACIEd was considered by the supervisors to be time-consuming, and leading to subjective interpretations and sometimes, conflicting assessments. The AAI was considered more relevant due to the “simplified” LO`s. However, AAI was thought to be difficult to use due to the Likert scale ranging from 1–7 were lack of clear definitions of the grading steps increased the complexity of assessing the LO`s.

#### Assessment

Supervisors described assessment from different perspectives as presented below in the following sub-themes: Summative Vs Formative assessment, Subjectivity in assessment, Pass/Fail or Likert scale, and Supervisors experience related to assessments. Within the sub-themes there were variations in how the supervisors described their experiences.

##### Summative Vs Formative assessment

The supervisors discussed if and how the assessment instruments could be used for formative or summative assessment and concluded that both instruments could be used for both purposes. However, the supervisors discussed whether ACIEd was harder to use as a formative instrument due to the sub-criteria in the LO`s and that the pass/fail scale were less useful then the 7 graded Likert scale when it came to displaying progress. The supervisors discussed whether displaying the student’s progression had a positive pedagogical value and they concluded as preferable relating it to formative feedback. Supervisors also discussed the frequency of formative assessments. Some supervisors raised concerns related to the increased workload generated by daily documented, formative feedback.

##### Subjectivity in assessment

To decrease the subjectivity, the supervisors discussed involving the patients in the assessments of students’ performance. They argued that the only persons who could assess the LOs concerning the patients’ own perception were the patients themselves. The supervisors concluded that more care encounters, with assessments between every encounter would probably produce a more accurate assessment of the student’s nursing skills. The supervisors also discussed the risk that the students would only be assessed according to the supervisor’s interpretation of what is a pass performance in relation to the assessment instrument.

The supervisors found that the assessments with a Likert scale could be beneficial but that the grading steps needed to be carefully defined to avoid subjective assessments. The definitions could be made clearer by using examples in relations to the grading steps and the LO`s where requirements could be listed for each step.

Supervisors said that assessments using a Pass/Fail grading was too definite. Supervisors described that assessing the care encounters became complicated since student’s performances may contain good and bad performances and with Pass/Fail grading the nuances did not become clear.

##### Supervisors experience related to assessments

Supervisors’ description of their relationship to the LO`s varied where some had more issues than others. Supervisors with less experience were more critical towards the LO`s then experienced supervisors. Furthermore, supervisors with less experience discussed that the student were obligated to display skills and knowledge and that supervisors were obligated to assess in accordance with the LO`s, meaning responsibility for demonstrating knowledge and skills fell on the student.

The more experienced supervisors argued that the LO`s were more like guidelines than specific goals to achieve, and that they used LO`s as a basis for discussions with the students. They argued that their clinical experience and understanding were the basis for the assessment, making the challenge with the LO`s less important, which contrasted with the view of the less experienced supervisors who interpreted the LO´s more literally. Furthermore, the more experienced supervisors discussed that complex care encounters offered scarce opportunities for the students to display knowledge and skills and that several care encounters were needed to assess knowledge and skills over time.

#### Supervisorship

The supervisorship was described by the supervisors in the following two sub-themes: Relationship with the students and Teams of supervisors.

##### Relationship with the students

All supervisors said that a relationship with the student could be a confounder when assessing students. They discussed whether a good relationship with the student would probably result in a more favourable assessment. The supervisors also claimed that a troubled relationship with the student might result in disinterest from their perspective which could result in diminishing feedback and lowered clinical education quality resulting in higher risk for failure. The supervisors discussed that formative feedback could help detect a lack of progression at an early stage and that measures could be taken to improve the situation for the student. Among the female supervisors a recurring statement was that it was difficult to assess the student negatively in the simulations because it felt harsh or even cruel to fail a student. Among the male supervisor’s similar feelings were described, but they related to the fact that assessments are not carried out on single occasions but over time, making the assessment instrument unfit for this kind of assessment. Male supervisors argued to a greater extent that it was hard to assess the student in the simulation due to lack of information about both patients and students. They argued that although some simulations were not as described in “textbook” examples, guidelines were bent daily and therefore it would be unfair to expect “textbook” care from the students. Hence, the male supervisors argued that the assessment instrument was a tool used for discussion and could not stand alone which it did in the simulations.

##### Teams of supervisors

The supervisors highlighted continuity as something important for the student’s learning, but it could also complicate their assessment since they may develop a relationship with the student. The desire for continuity in supervisorship could also create a problem when working schedules changed. This could result in the involvement of other supervisors, and disruption in the individual learning plan. The supervisors with longer experienced argued that it could be favourable with more than one supervisor involved in the assessment of the student, due to different perspectives. They argued that a supervisor team with different combinations of knowledge could be beneficial for the student, but that such teams must be coordinated and documented to ensure that the student’s learning progress was not hindered.

## Discussion

The findings showed that there were no significant differences in pass/fail gradings using the two different assessment instruments with the same content in the LOs, meaning that no matter what instrument was used the grade was the same when supervisors assessed the students in the simulated scenarios. However, there were significant differences within the assessment instruments, but the differences were consistent between the instruments, meaning that the quality of the assessment was considered equal. The differences within the assessment instrument can be explained by supervisor bias. Chong et al. showed in their study that seniority was a source for bias in LO`s related to communication but that the bias did not persist in LO`s related to physical examinations which is in line with the findings in this study [8]. The interviews with the clinical supervisors provided a more vivid picture of the complexity of assessments. Firstly, the intention of the assessments must be made clear for everyone. If the objective is formative assessment, the data indicates that using Likert scales is preferable to display progression. The complexity, phrasing and definition were recurring in the interviews, and it is a worth discussing why this is an issue. Supervisors rarely had any training in how to use the assessment instruments and as described, the more experience supervisors did not excel in their knowledge of the instruments but used their clinical experience to assess students from their own perspective. With this logic it would be wise to invest in training the supervisors rather than simplifying the LO´s. Prior research has shown that support from the supervisors during the clinical training is crucial to create a positive learning environment and to improve assessments [31].

However, the need for supervisor training and support does not mean that improving the assessment instrument is unnecessary. If a Likert scale is used, a clear definition of the grading steps is important as well as the layout of the instrument. As described by Immonen et al., it is important that the instrument is adapted to the every-day work, and fast and easy to use without losing its reliability and validity [14].

Subjectivity and complexity were reoccurring statements during the interviews. The supervisors highlighted this in all aspects regarding the clinical education. The supervisors’ own experience’ and expectations play a vital part in the clinical education as well as the relationship between the supervisor and the students which is supported in prior research [24]. To decrease subjectivity, an increase in the number of assessments made by more than one supervisor could be beneficial. Assessments after every care encounter in the ambulance service would generate a good basis for an overall assessment of the students’ performance. By organising the supervisors in teams, the different knowledges of the supervisors could be effectively utilized and possibly decrease subjectivity resulting in an improvement of the validity of the assessment. In contrast to this, prior research has shown that the relationship between the student and the supervisor is important to build trust and thereby a positive learning environment [30]. With documented, formative assessments accessible for both students and supervisors, continuity could be created through communication between supervisors. By using digital devices with LO`s and a Likert scale prepared in a mobile application, the assessments could be made easier to access, faster to use and the results could be displayed as a progression curve visible for students and supervisors. Digitalization of the assessment instrument also holds advantages concerning student possibility to argue for their grades. Without documented progress the students are in the hands of the supervisors as the only source of information about their performances. To further strengthen the validity of the assessments, other sources of feedback could be used. The supervisors discussed involving patients in the assessments and concluded that this could reduce subjectivity in the assessments and add other perspectives to the assessment. Further research is needed to fully understand the complexity of assessments and what methods to use to improve the quality of the assessment and strengthen the students learning.

### Methodological considerations

There are several limitations to this study. The number of supervisors included in the study was decided in discussion with the research team and a statistician after considering the availability of clinical supervisors and the extent of data needed for analysis. No power calculations were performed. Using standardised patients and simulation for assessing and grading students’ ability to care for patients may not reflect the clinical reality. However, since every care encounter is unique it would have been difficult to conduct a similar study in a clinical setting. Another limitation concerns the qualitative data collection. The interviewer is well known in the ambulance service, and this may have affected how the participants’ discussions concerning their experiences of using the assessment instruments in both a positive and negative way and increase the risk of bias. Conducting interviews between ambulance missions may also be considered as a limitation since the time for interviews was limited which could have resulted in participants not being able to develop their reasoning. The interviews were documented through field notes, and important information could have been missed. Field notes limit the possibility of quotations, which could have strengthened the validity of the findings in the interviews. Lastly, a limitation regarding the definition of experience needs mentioning. Experience in describe according to Benner but no data was collected regarding the supervisor’s experience of supervisorship. To supervise students is a natural part of the nursing profession but in hindsight, data concerning the quantity of students supervised during the supervisor’s clinical career would have offered clarity on the supervisor’s experience.

## Conclusion

The findings showed that there were no significant differences in pass/fail gradings using the two different assessment instruments containing the same LOs. However, the qualitative data suggests that supervisors struggled with subjectivity in the assessments as well as phrasing and definitions of the LO´s and the scales used in both instruments. This resulted in arbitrary assessments that were time-consuming and resulted in limited usage in the day-to-day assessment. The supervisors argued that the AAI was better adapted for formative assessment due to its Likert scale and simplified LOs, but a clear definition of the grading scales was considered important. Further research is needed concerning the validity of the assessments and how teams of supervisors can utilize different perspectives to improve the quality of the assessments. Digitalization could play a vital role in documenting feedback from multiple sources to enhance the formative feedback given to students during their clinical training. Transparency of documented feedback from multiple sources using a Likert scale provides an opportunity for the students to monitor their progress in situ. More research is needed to fully understand the mechanism behind the subjectivity of assessments and what methods could be used to strengthen the quality of the assessments and improve the quality of the clinical education.

### Supplementary Information

Supplementary Material 1.

Supplementary Material 2.

## Data Availability

The dataset generated and analysed in this study is available from the corresponding author on reasonable request.

## Abbreviations

AAI: Ambulance assessment instrument
ACIEd: Assessment of clinical education
LO: Learning objectives
OSCE: Observed structural clinical examination
RN: Registered nurses
