# Supplementary material for: Assessment during clinical education among nursing students using two different assessment instruments
Source: BMC Med Educ. 2024 Aug 7;24:852. doi: 10.1186/s12909-024-05771-x (PMC11308620; doi:10.1186/s12909-024-05771-x)
Supplement: Supplementary file 1 — Supplementary Material 1. [file 12909_2024_5771_MOESM1_ESM.docx]

**Appendix 1**

This appendix contains the learning objectives for the two assessment instruments Assessment of Clinical Education (ACIEd) and Ambulance Assessment Instrument (AAI). ACIEd is developed by Ulfarson and Oxelmark and is written in Swedish (Ulfvarson & Oxelmark, 2012). A translation of the learning objectives and its corresponding learning objectives are described below.

**ACIEd - Learning objective 1.**

1. Approach and support patients and their relatives in respectful consultation and perform nursing care based on the patient's experience of the situation.

**AAI - Learning objective 1.**

- 1. *To what extent was the patient treated with respect?*
  2. *To what extent was the patient allowed to describe his or her situation?*
  3. *To what extent did the student create a safe care situation?*

**ACIEd - Learning objective 2.**

1. Based on factual knowledge and the patient's experiences, observe, and carry out risk assessments of behaviors, signs, and symptoms in acute states of ill-health and disease, considering cultural aspects and the interaction between human and technology.

**AAI - Learning objective 2.**

2.1 *To what extent did the student display a good ability to observe and perform risk assessments of behaviors, signs and symptoms during acute ill-health and illness?*

2.2 *To what extent did the student display a good ability to use medical equipment in interaction with the patient?*

**ACIEd – Learning objective 3.**

1. Collaborate with the patient and/or relatives in the assessment of problems and needs, plan care and nursing, and analyze the consequences of prioritizations in complex situations.

**AAI - Learning objective 3.**

3.1 *To what extent did the student plan and priorities between nursing measures and the patient’s caring needs?*

3.2 *To what extent did the student analyze the consequences of priorities that was performed?*

***ACIEd – Learning objective 5.***

1. Reflect on the significance of one's own preunderstanding for equal treatment in encounters with the patient and relatives.

***AAI -* Learning objective 5.**

5. *To what extent did the student consider principles of equality when caring for an acutely ill patient?*

**ACIEd – Learning objective 9.**

*9.* Apply principles for a safe healthcare environment with a focus on hygiene and asepsis to prevent the spread of infections.

***AAI -* Learning objective 9.**

9. *To what extent did the student perform nursing measures in accordance with hygienic principles and aseptic methods?*

References

Ulfvarson, J., & Oxelmark, L. (2012). Developing an assessment tool for intended learning outcomes in clinical practice for nursing students. *Nurse Education Today, 32*(6), 703-708. doi:<https://doi.org/10.1016/j.nedt.2011.09.010>
