# Supplementary figures and images for: Assessment during clinical education among nursing students using two different assessment instruments
Source: BMC Med Educ. 2024 Aug 7;24:852. doi: 10.1186/s12909-024-05771-x (PMC11308620; doi:10.1186/s12909-024-05771-x)

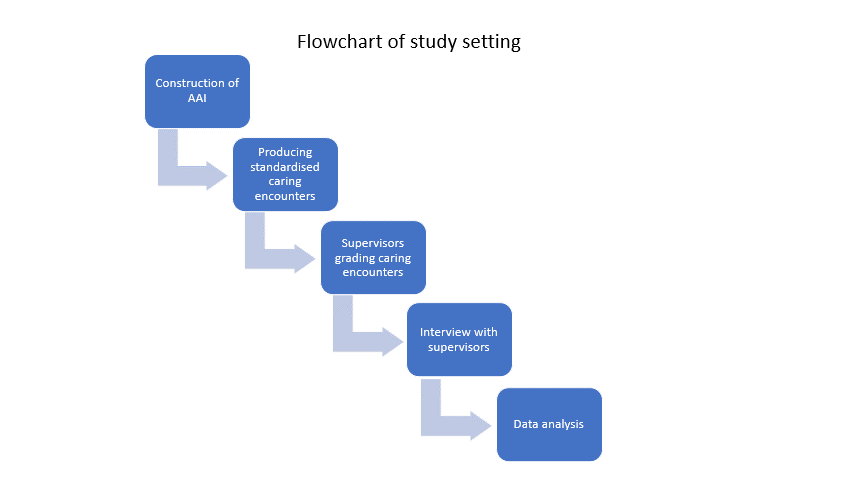

Supplement: Supplementary file 2 — Supplementary Material 2. [file 12909_2024_5771_MOESM2_ESM.gif]
